# Characterization of a New Mixture of Mono-Rhamnolipids Produced by *Pseudomonas gessardii* Isolated from Edmonson Point (Antarctica)

**DOI:** 10.3390/md18050269

**Published:** 2020-05-20

**Authors:** Carmine Buonocore, Pietro Tedesco, Giovanni Andrea Vitale, Fortunato Palma Esposito, Rosa Giugliano, Maria Chiara Monti, Maria Valeria D’Auria, Donatella de Pascale

**Affiliations:** 1 Institute of Biochemistry and Cell Biology, National Research Council, 80131 Naples, Italy; carmine.buonocore@ibbc.cnr.it (C.B.); tedesco@insa-toulouse.fr (P.T.); giovanniandrea.vitale@ibbc.cnr.it (G.A.V.); 2 Department of Marine Biotechnology, Stazione Zoologica Anton Dohrn, Villa Comunale, 80125 Naples, Italy; fpefortunato@gmail.com; 3 Department of Experimental Medicine, University of Campania “Luigi Vanvitelli”, 80138 Naples, Italy; rosa.giugliano@unicampania.it; 4 Department of Pharmacy, University of Salerno, 84084 Salerno, Italy; mcmonti@unisa.it; 5 Department of Pharmacy, University of Naples “Federico II”, 80131 Naples, Italy

**Keywords:** Antarctica, bioprospecting, rhamnolipid, antimicrobials

## Abstract

Rhamnolipids (RLs) are surface-active molecules mainly produced by *Pseudomonas spp.* Antarctica is one of the less explored places on Earth and bioprospecting for novel RL producer strains represents a promising strategy for the discovery of novel structures. In the present study, 34 cultivable bacteria isolated from Edmonson Point Lake, Ross Sea, Antarctica were subjected to preliminary screening for the biosurfactant activity. The positive strains were identified by 16S rRNA gene sequencing and the produced RLs were characterized by liquid chromatography coupled to high resolution mass spectrometry (LC-HRESIMS) and liquid chromatography coupled with tandem spectrometry (LC-MS/MS), resulting in a new mixture of 17 different RL congeners, with six previously undescribed RLs. We explored the influence of the carbon source on the RL composition using 12 different raw materials, such as monosaccharides, polysaccharides and petroleum industry derivatives, reporting for the first time the production of RLs using, as sole carbon source, anthracene and benzene. Moreover, we investigated the antimicrobial potential of the RL mixture, towards a panel of both Gram-positive and Gram-negative pathogens, reporting very interesting results towards *Listeria monocytogenes* with a minimum inhibitory concentration (MIC) value of 3.13 µg/mL. Finally, we report for the first time the antimicrobial activity of RLs towards three strains of the emerging multidrug resistant *Stenotrophomonas maltophilia* with MIC values of 12.5 µg/mL.

## 1. Introduction

Among glycolipids, rhamnolipids (RLs) are the best-known and characterized biosurfactants [1], and consist of either one or two rhamnose units linked by a ß-glycosidic bond with a 3-(hydroxyalkanoyloxy) alkanoic acid (HAA) fatty acid tail ranging between 8 and 16 carbons in length [2]. So far, more than one hundred RL homologues have been discovered, and they differ from each other mainly in the length of the fatty acid chains and in the degree of unsaturation [3,4]. RLs are involved in motility, enhancing and modulating the swarming movement [5], in the improvement of the uptake of the poorly soluble hydrocarbons [6], in the biofilm formation and structure [7], and antimicrobial activities, such as antibacterial, antifungal, and antialgal [8,9,10,11]. In particular, these compounds showed strong activity towards a wide range of bacteria and fungi [12,13,14].

Cold environments are defined as places permanently exposed to temperatures below 5 °C and account for more than 80% of the Earth’s biosphere. The polar regions represent nearby 15% [15] and could represent a huge resource of unexplored natural products, in particular biosurfactants. Indeed, microorganisms living in the cold environment have shown enhanced biosurfactant production to cope with cellular and proteins disruption, due to the ice and water phases cycles, and the carbon sources limitations [16,17]. Antarctica is the coldest and most largely unexplored place on Earth and hosts microbes able of withstanding high selective pressures, such as high UV-radiation, drought, light limitation and extremely low temperatures [18,19]. Therefore, thanks to its selective living conditions, Antarctica harbors many strains with valuable features for biotechnology and therefore, bioprospecting represents a promising strategy for the isolation of strains capable to produce new molecules of interest.

In a previous study, our research group described the isolation and the identification and characterization of two novel RLs produced by a strain isolated from Antarctic shallow water. These RLs showed antimicrobial activity towards *Burkholderia cepacia* complex, a group of opportunist multidrug resistant human pathogens, suggesting a potential role of these molecules in the fight against multi drug resistant bacteria [20].

In this study, the production of RLs by Antarctic bacteria has been investigated. The study involved the isolation of microorganisms, selection of the active strains by three complementary rapid screens for the biosurfactant activity, phylogenetic affiliation of the active strains by 16S rRNA sequencing, cultivation, solvent extraction of metabolites, 16S rRNA sequencing and LC-HRESMS identification of the compounds. Moreover, the influence of the carbon source on the mixture complexity and its antimicrobial ability was also investigated.

## 2. Results and Discussion

### 2.1. Bacterial Isolation and Biosurfactant Activity Screening

Isolation of bacteria from sediments collected from Edmonson Point was performed on marine agar (MA) and TYP agar plates. After 15 days of incubation at 20 °C, 34 morphologically different colonies were selected, 21 from MA plates and 13 from TYP plates.

**Figure 1:**
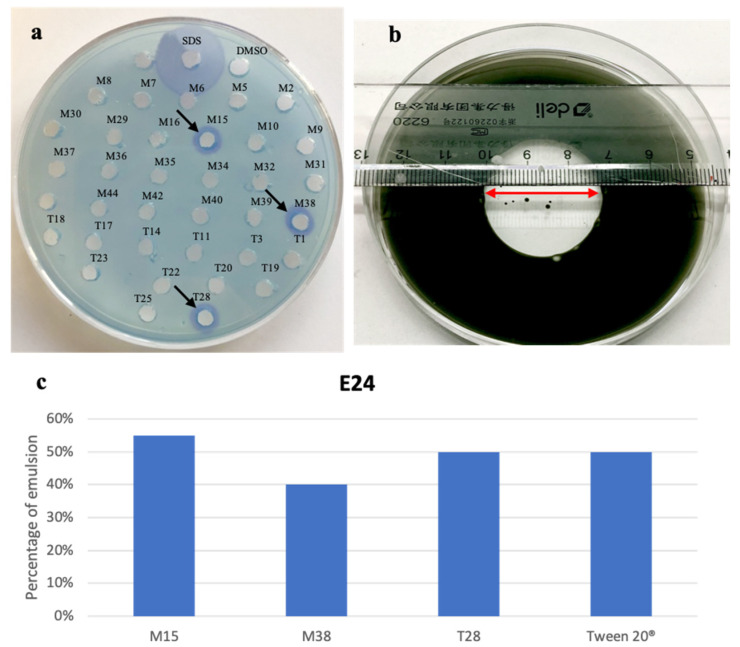
(**a**) Biosurfactants activity on a cetyltrimethylammonium bromide (CTAB) agar plate, the blue halos indicate the positivity to the test, the arrows show the positive extracts; (**b**) oil spreading test, the red arrows indicate the diameter of the halo; (**c**) the graph shows the E24 values of the tested supernatants and Tween 20^®^.

All the strains were cultured in the respective liquid media. After 5 days of incubation, the culture broths were centrifuged and the supernatants were extracted by ethyl acetate. The obtained crude extracts were screened for biosurfactant activity. Only 3 strains (M15, M38, T28) exhibited positivity to the biosurfactant activity tests, out of 34 bacterial isolates (Figure 1).

The emulsification indexes (E24) [21], the oil spreading in water [22] and the reactions with dyes such as cetyltrimethylammonium bromide (CTAB) [23] were used as complementary assays to detect the production of anionic biosurfactants [24].

The presence of anionic biosurfactants in the extracts of M15, M38 and T28 was confirmed by the arising of a dark blue halo in the CTAB agar plate (Figure 1a). After 48 h at 4 °C, the diameters of the dark blue haloes were detected: M15 (ø 0.88 cm), M38 (ø 0.87 cm), T28 (ø 0.89 cm). SDS 0.1%, positive control showed a 2.16 cm diameter halo. CTAB agar test is specific for anionic biosurfactants, thus, in order to investigate the presence of other biosurfactants in the crude extracts, the oil spreading test was performed. This assay can reveal the presence of biosurfactants through the development of a clear halo in the oil-water surface. Again, the best results were obtained by M15 (ø 2.6 cm) (Figure 1b), while M38 and T28 gave (ø 1.8 cm) and (ø 2.4 cm), respectively. The assessment of E24 (55%, 40%, 50% and 50%, respectively, Figure 1c) using Tween 20^®^ as a positive control indicated a remarkable emulsifying power towards n-Hexane.

### 2.2. Bacterial Identification

**Figure 2:**
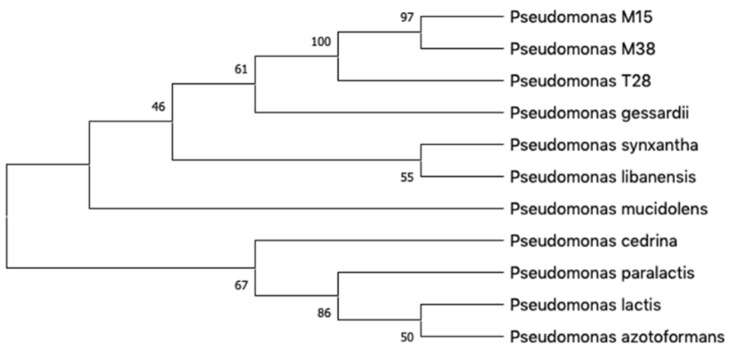
Phylogenetic tree generated with MEGAX based on 16S rRNA gene sequences of M15, M38 and T28 strains and related species. Next to the branches are shown the percentage of replicate trees in which the associated taxa clustered together in the bootstrap test.

To identify M15, M38 and T28, 16S rRNA amplicons were sequenced and investigated through EzBioCloud [25]. The results were utilized to build a phylogenetic tree with a set of related species (Figure 2). The output of both EzBioCloud and the phylogenetic tree showed that the investigated strains were closely related, if not conspecific, to *P. gessardii* DSM 17152 [26], with values of similarity and variation ratio, respectively, of 99.43% and 8/1412 bp for M15, 99.78% and 3/1344 bp for M38, and 99.93% and 1/1351 bp for T28. *P. gessardii* belongs to the *P. fluorescence* group that was already reported to produce rhamnolipids [27]; moreover, a strain closely related to *P. gessardii* and able to produce RLs was recently isolated in Antarctica [28].

Therefore, giving the close phylogenetic relationship between the three selected strains, only the most active M15 was selected for further investigation.

### 2.3. Chemical Characterization

The crude ethyl acetate extract was subjected to first fractionation by solid-phase extraction (SPE) with C-18 cartridges as described in Materials and Methods. In order to confirm the activity, the obtained fractions were subjected once again to CTAB agar and oil-spreading assay. Fractions eluted at 80% and 100% methanol (MeOH) showed positivity to both assays. No activity was shown by the fraction eluted at 60% MeOH.

**Figure 3:**
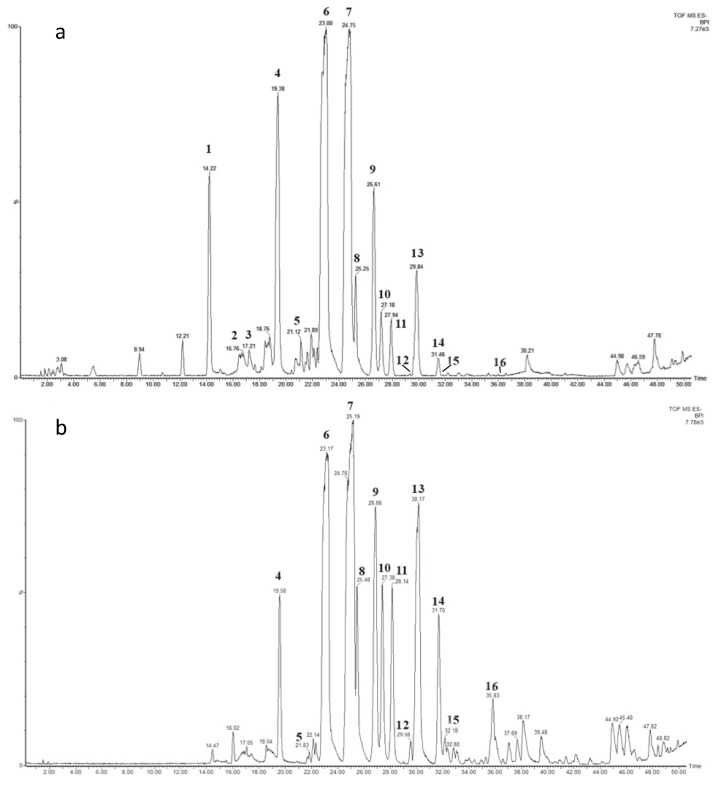
Total ion chromatogram of: (**a**) M15 SPE 80% and (**b**) M15 SPE 100% fractions. The rhamnolipids (RLs) peaks are numerated and shown in Table 1.

The 80%- and 100%-MeOH fractions, both positive to the biosurfactant activity tests, were subjected to LC-HRESMS dereplication in both positive and negative modes. The total ion chromatograms recorded in negative mode (Figure 3) of both fractions displayed a similar pattern, indicating a complex mono-rhamnolipid mixture. As extensively reported in the literature [29,30,31], the analysis of the negative pseudo-molecular ion [M − H]^−^ obtained by LC-HRESMS gave information on the molecular formula. Under the experimental conditions used for the analysis, the primary ion underwent spontaneous in-source fragmentation, giving rise to the key fragment ion arising from the cleavage of the ester linkage between the two ß-hydroxy fatty acid units, allowing the discrimination between congeners with non-symmetric fatty acid units. For instance, the molecular formula C_24_H_44_O_9_ for the peak at 14.22 min (Figure 3a) corresponds to a Rha-C_8_-C_10_ or Rha-C_10_-C_8_ structure. The key fragment at m/z 305.1290, observed in the mass spectra (S1. Supplementary Materials) corresponding to Rha-C_8_, allowed us to assign the Rha-C_8_-C_10_ structure.

Mass spectra dereplication highlighted the presence of a total of 16 different mono-RLs, about half of them displayed the presence of at least one unsaturation in one of the fatty acid (FA) chains (Table 1). Compounds 3, 8, 12, 14 and 15 are described for the first time in this work, while the other are already present in literature [32,33].

**Figure 4:**
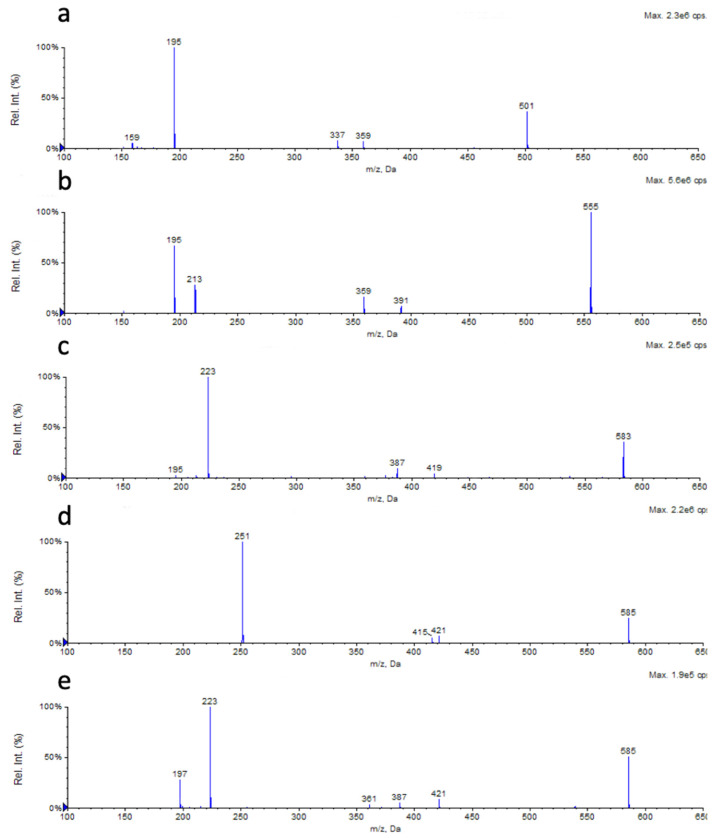
The MS/MS spectra of the new RLs (**a**) Rha-C_12:1_-C_8_, (**b**) Rha-C_12:1_-C_12:1_, (**c**) Rha-C_14:1_-C_12:1_, and (**d**) Rha-C_16:1_-C_10_ confirmed their structure predicted on the basis of their in-source fragmentation. (**e**) The MS/MS spectra of the compound under the peak 15 showed the presence of the two RLs Rha-C_14:1_-C_12_ and Rha-C_12_-C_14:1_.

The fragmentation of the [M − H]^−^ adducts of the five new RLs led to three key ions for each one, Rha-FA1, FA1-FA2 and FA2, which further confirmed the previously hypothesized structure for the compounds 3, 8, 12 and 14 (Figure 4a–d). The compound 15, submitted to MS/MS fragmentation, gave the simultaneous presence of the daughter ions 223, 361 and 421, respectively, due to C_12_, Rha-C_12_ and C_12_-C_14:1_, together with the ions derived from the fragmentation of Rha-C_14:1_-C_12_ (Figure 4e), highlighting the co-occurrence of the two structural isomers, Rha-C_14:1_-C_12_ and Rha-C_12_-C_14:1_.

These molecules are all characterized by the presence of an unsaturation on the FA1 chain, the compounds 8 and 12 also displayed an additional unsaturation on FA2 chain. *P. gessardii* has been reported in the literature for the production of biosurfactants such as RLs [28] and a lipoprotein [34]. In particular, Kristoffersen et al. [28] reported the production of five mono-rhamnolipids with the same formula of compounds 4, 6, 7 and 9 found in this work (Table 1). 

### 2.4. Analysis of the Influence of the Carbon Source on the RL Chemical Composition

The preference in the carbon source is strain-dependent and may greatly influence the RLs composition [35,36,37,38,39]. Moreover, carbon source has a great impact also in the industrial production economy of the RLs, as it accounts for the 10–30% of the total costs for recombinant production process [40]. Therefore, the capability of a strain to use using low cost or waste materials such as glycerol and used cooking oil (UCO) represent a great advantage. Glycerol is a byproduct of biodiesel industry and its market cannot accommodate the amount generated [40], UCO is a kitchen-generated waste that causes serious environmental problems. When collected, it is utilized mainly in biodiesel production, but can find utilization as a carbon source in fermentation process [41].

M15 strain was grown in the presence of different carbon sources, in order to explore as them affect the RLs mixture composition. Moreover, the ability to produce RLs from petroleum derivate hydrocarbons, both aromatic and aliphatic, as sole carbon source was also explored in a bioremediation context.

Phosphate-limited peptone-ammonium salt (PPAS) medium was utilized as minimal medium, as modification of PPGAS (phosphate-limited peptone-glucose-ammonium salt) [5], and after the incubation, the cell-free broths were extracted and subjected to LC-MS analysis to investigate the presence of RLs.

Isolate M15 was able to utilize all the tested carbon sources for growth while no growth was detected when cultivated in PPAS not supplemented with a carbon source. However, the composition of the RLs mixture was greatly affected by the different carbon sources.

To evaluate these changes in the composition, base peak chromatograms were manually integrated and the relative abundance of the single RL peak, in the RLs mixture, was calculated for each growth condition (Table 2). TYP crude extract (CE) was used as reference condition, detecting RLs 6 and 7 as the most abundant with more than 20% each, followed by RLs 1, 4, 9, and 13 with a relative abundance of near 10%. RLs 8, 10, and 11 accounted for less than 4%, RLs 14 and 16 for less than 2%, while all the others RLs were less than 1% of the total composition.

Among monosaccharides, glucose and mannose gave better results than rhamnose in term of numbers of congeners production, but the relative abundance of the single RLs was very dissimilar. In fact, in mannose, the RLs 1, 6 and 7 accounted for more than 80% of the entire mixture, while in glucose the same compounds were less than 60%. Out of the three monosaccharides, rhamnose gave the worst results in terms of RL congeners production; in fact, eight RLs were completely missing or drastically reduced, while the presence of compounds 2 and 6 in the mixture were quadrupled and doubled, respectively. The polysaccharides starch and xylan gave very similar results, with a difference represented by compounds 10–13, which are not present in xylan, while in starch were present three times more than TYP CE.

Glycerol showed the presence of all the RLs and together with UCO exhibited the highest relative abundance of the new RLs. In particular, compound 3 in glycerol and UCO was ten and six times more abundant than in TYP CE, respectively. RLs 8, 14 and 15 were doubled in both conditions, while the abundance of RL 2 was more than quadrupled in UCO.

Considering petroleum derived carbon sources, diesel gave an interesting mixture, in which the quadrupled production of RLs 2 and 3 and the lack of 15 and 16 were the most significant results. The relative composition of the benzene was afflicted by the lack or drastic reduction in RLs 5, 10, 11, 13, 14, 15, and 16, while the quadrupled abundance of compound 12 was notable. Among polyaromatic hydrocarbons (PAH), anthracene gave remarkable results, showing the presence of 11 RLs on a total of 16. To the best of our knowledge, benzene and anthracene were reported here for the first time as the sole carbon source in RLs production. Finally, the production of RLs was not detected in pyrene, while on phenanthrene, only RL 6 (Figures S17–S29) was found. Singh and Tiwary reported the use of these two PAHs as the sole carbon source for glycolipids production from *Pseudomonas otitidis*, but the glycolipids class was not specified [42].

Glycerol and UCO gave the best results in congeners production. This can be explained through consideration on the availability of the RLs precursors, L-rhamnose and 3-hydroxyalkanoate. Thanks to metabolism flexibility, these two metabolites can be produced from many carbon sources, such as sugars, vegetable oils, glycerol, and hydrocarbons, although with a different metabolic cost for the cells [43]. However, fatty acids can be directly incorporated in the lipidic chains of RLs and this can explain the higher number of congeners when the strains are using UCO as a carbon source. On the other hand, glycerol was already reported as a good soluble carbon source for RL production, since it could act as a close biosynthetic precursor of both lipid and sugar building blocks in RL synthesis [44].

RLs are well-known for their bioremediation potential and they have been proven to help in PAH degradation by reducing their hydrophobicity and enhancing their biodegradation by the microbial community [45,46]. Considering M15 capability of producing RLs from pollutants such as anthracene, benzene and diesel, this strain proves to be a suitable candidate for bioremediation applications.

### 2.5. Antimicrobial Activity

The M15 SPE fractions were evaluated for their antimicrobial activity by liquid inhibition assays towards 17 human pathogen bacteria (Table 3). The fraction eluted at 60% of MeOH did not show activity. On the other hand, fractions eluted at 80% and 100% of MeOH were shown to be active towards the majority of the tested Gram-positive bacteria. This evidence is in accordance with the literature [47,48] and can be explained by the ability of biosurfactants to disrupt membrane structure disturbing interactions with phospholipids and membrane proteins of Gram-positive bacteria [49]. Both TYP 80% and 100% fractions showed very low MIC values towards *B. cereus*, *L. monocytogenes*, *S. aureus* strains and *S. epidermidis* with values in that vary from 6.25 to 25 µg/mL for the former and from 3.13 to 25 µg/mL for the latter. Differently from others Gram-positive bacteria, *S. xylosus* showed no sensitivity in the tested concentrations, while *S. epidermidis* showed low sensitivity, with MIC values of 50 and 100 µg/mL for the 80% and 100% fractions, respectively.

Although antimicrobial activity of RLs towards Gram-negative bacteria is not uncommon [20,50], generally these bacteria are resistant to anionic surfactants because their outer membrane is hardly permeable to hydrophobic and amphipathic molecules [48,51,52]. Despite *A. baumannii*, *B. metallica*, *E. coli*, *K. pneumoniae*, *P. aeruginosa*, *S.* Enteritidis and *S.* Typhimurium being resistant to the fractions in the tested concentrations, *S. maltophilia* strains showed high sensitivity to them, with a MIC value of 12.5 µg/mL for the 80% fraction and 25 µg/mL for the 100% towards strains 700475 and 13636, while strain 13637 showed higher sensitivity, with a MIC value of 12.5 µg/mL for both fractions. This is a very promising finding, as *S. maltophilia* possesses high level of intrinsic resistance to many antimicrobials and it is also readily able to acquire multidrug resistance when exposed to different antibiotics [53,54]. To the best of our knowledge, this is the first report of a mixture of mono-RLs displaying activity towards *S. maltophilia*.

All the tested pathogens showed sensitivity only to the fractions containing RLs and, although there are some minority unidentified compounds in the fractions, we can speculate that these molecules could be responsible for the antimicrobial activity.

As the antimicrobial activity towards the different strains of both *S. aureus* and *S. maltophilia* were similar, the antimicrobial activity of glucose, mannose, rhamnose, glycerol, and TYP CE extracts were evaluated towards a restricted panel of pathogens, such as *B. cereus*, *L. monocytogenes*, *S. aureus 23235*, *S. epidermidis*, *S. maltophilia 13637* (Table 4). As reported, different factors affect the antimicrobial activity of both RLs and RLs mixture, such as the congeners composition, the length of the acyl chains, and the presence of unsaturation [20,28,50]. The relative RL composition of glucose, mannose rhamnose, glycerol, xylan, starch and TYP CE extracts (Table 2) was matched with their MIC values (Table 4), allowing us to link the antimicrobial power to a restricted range of congeners. The variations in both the RLs mixture composition and in antimicrobial activity were compared to the TYP CE that was utilized as a reference. The best antimicrobial activities were obtained by the glycerol and two TYP SPE 80% and 100% fractions extracts, which showed the highest relative abundance in the mixture of RLs 8 and 9, and of RLs from 10 to 16. This evidence was particularly highlighted on glycerol, in which relative abundances of RL 8, RL 9 and RL 14, compared to TYP CE, were doubled, 1.5 times more and nearly tripled, respectively. On the contrary, the abundance of RLs 8, 9 and 14 was drastically reduced in mannose and rhamnose that showed low and absent antimicrobial activity, respectively, while they are nearby absent in starch and xylan that showed no activity. This might suggest that RLs 8, 9 and 14 could be the major RLs responsible for the antimicrobial activity. Moreover, the antimicrobial activity of 9 was already reported in the literature [28], while compound 8 and 14 were reported here for first time along with their bioactivity.

## 3. Materials and Methods

### 3.1. Isolation of Microorganisms

Bacterial strains were isolated from sediments collected in Edmonson Point Lake, Ross Sea, Antarctica, 74° 20′ (74.3333°) South, 165° 8′ (165.1333°) East. To obtain a cells suspension, 1 g of sediments was mixed with 20 mL of M9 salts solution in a 50mL Falcon sterile tube and gently mixed. The suspension was homogenized using a vortex, serially diluted (10^−1^, 10^−2^ and 10^−3^ in 10 mL of M9) and 100 µL of each dilution was plated on MA and TYP agar and incubated at 20 °C for 15 days. After the incubation period, morphologically different colonies were picked, grown in liquid marine broth (MB) and TYP and stored at −80 °C.

### 3.2. Media and Buffers

The following media and buffers were used during this study:

**M9 salts** (3 g/L KH_2_PO_4_, 6 g/L Na_2_HPO_4_, 5 g/L NaCl, 1 mL 1 M MgSO_4_); **MB** (19.4 g NaCl, 8.8 g/L MgCl_2_, 5 g/L peptone, 3.24 g/L Na_2_SO_4_, 1.8 g/L CaCl_2_, 1 g/L yeast extract, 0.55 g/L KCl, 0.16 g/L NaHCO_3_, 0.10 g/L Fe(III) citrate, 0.08 g/L KBr, 0.034 g/L SrCl_2_, 0.022 g/L H_3_BO_3_, 0.008 g/L Na_2_HPO_4_, 0.004 g/L sodium-silicate, 0.0024 g/L NaF, 0.0016 g/L NH_4_NO_3_); **TYP** (16 g/L bacto-tryptone, 16 g/L yeast extract, 10 g/L NaCl); **lysogeny broth (LB)** (10 g/L tryptone, 5 g/L yeast Extract, 10 g NaCl); **PPAS** (10 g/L peptone, 0.1 g/L MgSO_4_, 1.09 g/L NH_4_Cl, 1.5 g/L KCl, 18.9 g/L tris base, pH adjusted to 7.2 with HCl).

### 3.3. Extract Preparation

A single colony of a bacterial isolate was used to inoculate 3 mL of liquid MB or TYP media in a sterile bacteriological tube. After 48 h of incubation at 20 °C at 210 rpm, the pre-inoculum was used to inoculate 125 mL of the same medium in 500 mL flasks at an initial cell concentration of 0.01 OD_600_. The flasks were incubated up to 5 days at 20 °C at 210 rpm. The cultures were then centrifuged at 6800 × g at 4 °C for 45 min, the cell-free culture broths were collected and subjected to organic extraction twice with 2 volumes of ethyl acetate, in a 500 mL separatory funnel. The organic phase was collected and evaporated using a rotavapor (R-100, BUCHI, Flawil, Switzerland) and the extracts were weighted, dissolved in 100% DMSO at the concentration 100 mg/mL and stored at −20 °C.

M15 strain was also grown in PPAS supplemented with 12 different carbon sources at 1% *w*/*v* final concentration, such as glucose, mannose, rhamnose, starch, xylan, benzene, diesel, anthracene, pyrene, phenanthrene, glycerol, and UCO. After 5 days of incubation, 14 for PAHs, the cell-free culture broths were collected, and extractions were performed as described above.

### 3.4. Biosurfactant Screening

The presence of biosurfactants in the extracts was investigated by means of three tests, one carried out on 90 × 15 mm plate (CTAB agar plate method) and two in liquid (oil spreading test and emulsification capacity assays).

#### 3.4.1. CTAB Agar Method

The CTAB agar method, also called Blue agar, is an in-plate test that can reveal the presence of anionic biosurfactants by the arising of dark blue halos around the extracts. In this method, the anionic biosurfactants form an insoluble complex with cetyltrimethylammonium bromide, and the complex is revealed by the presence of methylene blue. Wells were made in the agar, with the wide top of a sterile Pasteur pipette, and filled with 8 µL of the extracts, dissolved in DMSO at 100 mg/mL. As a negative control, 8 µL of pure DMSO were used, while 8 µL of 0.1% sodium dodecyl sulphate (SDS) were used as positive control. After 2 days at 4 °C, the extracts containing RLs were selected by the presence of a dark blue halo around the wells. The halo diameter was directly proportional to the surfactant concentration [2].

#### 3.4.2. Emulsification Capacity Assay

This test depends on the ability of biosurfactants to stabilize emulsions. The method was performed adding to 1 mL of n-hexane, 1 mL of free-cell culture supernatant in 6 mL glass tubes (7.5 cm × 1 cm) and vortexing for 2 min. Tween 20^®^ (0.5% *v*/*v*) was used as positive control. After 24 h of incubation at room temperature, emulsification capacity was optically determined as a stable emulsion. Moreover, the E24 was calculated as the percentage of the height of the emulsified layer (mm) divided by the total height of the liquid column.

(1) $$E24\left(\%\right)=\frac{Emulsified\;layer\;height\;\left(mm\right)}{Total\;liquid\;height\;\left(mm\right)}\times 100$$

#### 3.4.3. Oil-Spreading Test

The test was developed by Morikawa et al. [22] and can reveal the presence of biosurfactants by the solubilization of crude oil in water. In detail, biosurfactants can solubilize oil in water by micelles formation making a clear zone into the oil layer.

A thin oil layer on the water’s surface was made adding 50 µL of exhaust motor oil to 25 mL of distilled water in a Petri dish. Then, 1 µL of crude extract at 1 mg/mL was delivered onto the oil. DMSO was used as a negative control.

### 3.5. Bacterial Identification

The identification of strains positive for both biosurfactant and antimicrobial screenings were carried out amplifying 16S rRNA gene using a single colony as template. PCR was carried out in a total volume of 50 µL, containing 25 µL of DreamTaq PCR Master Mix (a ready-to-use solution containing DreamTaq DNA Polymerase, optimized DreamTaq buffer, MgCl2, and dNTPs), 0.2 µM of both primer 27F (Forward, seq: 5’-AGAGTTTGATCCTGGCTCAG-3’) and 1492R (Reverse, seq: 5’-GGTTACCTTGTTACGACTT-3’). The reaction conditions used were: one cycle (95 °C for 10 min), 30 cycles (95 °C for 30 s, 50 °C for 30 s, and 72 °C for 2 min), with a final extension of 7 min at 72 °C. PCR products were then purified by GenElute^TM^ PCR Clean-UP kit (Sigma-Aldrich, Darmstadt, Germany), the purified PCR products were sequenced by Microgrem (Napoli, Italy). Both end sequences obtained by submitting the forward and the reverse to Prabi CAP3 [55] (http://doua.prabi.fr/software/cap3) were submitted to EzBioCloud for the affiliation analysis. Evolutionary analyses were conducted in MEGA X [56]. A phylogenetic tree was inferred using the neighbor-joining method [57]. The evolutionary distances were computed using the Kimura 2-parameter method [58] and were in the units of the number of base substitutions per site. All positions with less than 95% site coverage were eliminated, i.e., fewer than 5% alignment gaps, missing data, and ambiguous bases were allowed at any position (partial deletion option). Bootstrap values were calculated with 1000 resamples.

### 3.6. Biosurfactant Fraction Enrichment

The extract obtained from a 2 L culture of strain M15 in liquid TYP was re-suspended in the minimum possible amount of MeOH and subjected to fractionation using C18 cartridges (Macherey-Nagel, Duren, Germany), utilizing H_2_O, MeOH and mixtures of the two in different percentages as eluents. Fractions eluted at 60%, 80% and 100% of MeOH were collected, dried and tested using the liquid antimicrobial assay and oil-displacement test.

### 3.7. Chemical Profiling and Structural Analysis of Biosurfactants

ESI-MS spectra were carried out in negative mode on a high-resolution mass spectrometer QToF Premiere (Waters Corp., Manchester, UK) equipped with Alliance 2610 pumps. The following parameters were set for MS: Capillary (kV) 3.2; Sampling Cone 40.0; Extraction Cone 3.0; Ion Guide 2.0, Collision Energy 5.0. The extracts were dissolved in MeOH at 12 mg/mL and 3 µL, ca 20 µg, were injected in a Kinetex reverse C18 column (Phenomenex, Torrance, CA, USA). The gradient was run at a flow of 200 µL/min, using H_2_O and MeOH, respectively, as solvent A and solvent B, and to both 5 mM ammonium acetate was added. The gradient went from 50% to 95% B in 45 min.

The MS/MS experiments, different carbon sources ESI-MS, and both TYP CE and fractions ESI-MS were conducted on a QTRAP 4500 (SCIEX, Framingham, MA, USA), with the ESI source in negative mode connected to a Nexera X2 UHPLC (Shimadzu, Kyoto, Japan), equipped with a ACQUITY UPLC BEH 2.1 × 50 mm C18 column 1.7 µm (Waters, Milford, MA, USA). The solvent system consists of mass grade solvents, (A) water containing 0.1% formic acid and (B) acetonitrile containing 0.1% formic acid. The gradient was programmed as follows: from 25% to 80% B in 60 min, from 80% to 100% in 1 min, 100% B isocratic for 7 min, from 100% to 25% B in 1 min and finally, the initial conditions were held for 3 min as a re-equilibration step. The flow rate was 0.2 mL/min, the injection volume was 3 μL, and the extracts were dissolved in MeOH at 10 mg/mL. The mass spectrometry conditions were as follows: source temperature 250 °C, capillary voltage −4.5 kV, range m/z 100–650. MS/MS conditions were as follows: collision energy at −35 ± 15 eV collision energy (CE) was used to fragment ions in the m/z 100–650 range.

The area of the RL peaks detected by LC-ESI-MS experiments carried out on QTRAP 4500 were obtained through the script “Manually Integrate” of Analyst^®^ software (SCIEX, Framingham, MA, USA). Relative abundance of each peak was calculated as the percentage of the single RL peak divided by the sum of all the RLs peaks areas.

(2) $$Relative\;abundance=\frac{RL\;area}{Total\;RLs\;areas}\times 100$$

### 3.8. Antimicrobial Activity

The SPE fraction, enriched in RLs, were tested for their antimicrobial activity microtiter plates assay. The extracts were placed into each well of a 96-well microplate at an initial concentration of 200 µg/mL and serially 2-fold diluted using LB medium. A control for external contaminations was represented by wells containing only the medium. DMSO (2% *v*/*v*) was used as negative control to determine the effect of the solvent on bacterial growth. A single colony of each pathogen strain was used to inoculate 3 mL of liquid medium in a sterile 13 mL tube. After 5–8 h of incubation, growth was measured by monitoring the absorbance at 600 nm and about 40000 CFU were dispensed into each well of the prepared plate. Plates were incubated at 37 °C. The absorbance of the 96-well plates was measured at 600 nm at time zero and after an overnight growth, by ELX800 Absorbance Microplate Reader (Biotek, Winoosky, VT, USA), in order to evaluate the growth of the pathogens. To evaluate the antimicrobial capacity of the extracts, a panel of model multidrug resistant pathogens were used: *Acinetobacter baumannii* Ab13 [59], *Bacillus cereus* ATCC 14579 [60], *Burkholderia metallica* LMG 24068 [61], *K. pneumonie* DF12SA [62], *E. coli* ATCC 10536 [63], *Listeria monocytogenes* MB 677 [64], *Pseudomonas aeruginosa* PA01 [65], *Salmonella enterica* serovar Enteritidis ATCC 13076 [66], *Salmonella enterica* serovar Typhimurium MB 4487 (ILVO), *Staphylococcus aureus* ATCC 29213 [67], *Staphylococcus aureus 23235* [68], *Staphylococcus aureus* 6538P [69], *Staphylococcus epidermidis* ATCC 35984 [70], *Staphylococcus xylosus* MB 5209 [71], *Stenotrophomonas maltophilia* ATCC 13637 [72], *Stenotrophomonas maltophilia* ATCC 13636 [73] *Stenotrophomonas maltophilia* ATCC 700475 [74]. All pathogens were grown overnight in LB at 37 °C with orbital shaking at 210 rpm.

## 4. Conclusions

In this study, we isolated from Antarctic sediments a strain identified as *P. gessardii* and able to produce biosurfactants. LC-HRESMS analysis revealed the presence of 17 different mono-RLs in the extract. Structural analysis by LC-MS/MS revealed that six of them, Rha-C_12:1-_C_8_, Rha-C_12:1_-C_12:1_, Rha-C_14:1_-C_12:1_, Rha-C_16:1_-C_10_, Rha-C_14:1_-C_12_, and Rha-C_12_-C_14:1_, were never described before. We also investigated the ability of the strain to grow and produce RLs from cheap carbon sources and pollutants reporting the relative abundance of the singles RLs in each condition. The best results, in terms of relative abundance of the new RLs, were obtained from glycerol, oil, mannose and glucose. RL production obtained from diesel, benzene and anthracene was a remarkable result from a bioremediation point of view. Thus, M15 is able to use PAHs as a sole carbon source to grow and to produce RLs that help to degrade PAHs by the local microbial community. We also evaluated the antimicrobial potential of the whole RL mixtures, obtaining interesting results against *B. cereus*, *L. monocytogenes* and *S. aureus* and reporting for the first-time antimicrobial activity of RLs towards *S. maltophilia*. We also correlated the increments of the antimicrobial activity to Rha-C_12:1_-C_12:1_, Rha-C_14:1_-C_10_ and Rha-C_16:1_-C_10_, through their relative abundance in the RL mixtures. Moreover, we highlighted the overproduction of the new RLs on glycerol and UCO, a finding that could be helpful in the future perspectives of isolation and purification of these compounds.

## Figures and Tables

**Table 1:** Assignment of the identified RLs present in the LC-MS TICs of M15 80% and 100% fractions (Figure 3) ^a^.

No.	Retention Time (min)	Measured [M − H]^−^ (m)/z	Δ ppm vs. Theoretical Value	Molecular Formula	Key Fragment (Rha-FA1)	RL	n_1_	n_2_	Structure
**1**	14.22	475.2779	−26.9	C_24_H_44_O_9_	305.1290	Rha-C_8_-C_10_	1	3	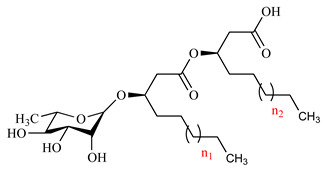
**2**	16.76	489.3087	+4.7	C_25_H_46_O_9_	319.1520	Rha-C_9_-C_10_	2	3	
**3**	17.21	501.2946	−23.5	C_26_H_46_O_9_	359.2195	Rha-C_12:1_-C_8_	5(-2H)	1	
**4**	19.38	503.3036	−36.6	C_26_H_48_O_9_	333.1660	Rha-C_10_-C_10_	3	3	
**5**	21.12	517.3278	−12.1	C_27_H_50_O_9_	381.2199	Rha-C_11_-C_10_	4	3	
**6**	23.00	529.3102	−52.0	C_28_H_50_O_9_	359.1872	Rha-C_12:1_-C_10_	5(-2H)	3	
**7**	24.75	531.3319	−40.3	C_28_H_52_O_9_	361.2039	Rha-C_12_-C_10_	5	3	
**8**	25.26	555.3454	−14.2	C_30_H_52_O_9_	359.1942	Rha-C_12:1_-C_12:1_	5(-2H)	5(-2H)	
**9**	26.63	557.3553	−24.6	C_30_H_54_O_9_	387.2223	Rha-C_14:1_-C_10_	7(-2H)	3	
**10**	27.17	557.3553	−24.6	C_30_H_54_O_9_	361.2039	Rha-C_12_-C_12:1_	5	5(-2H)	
**11**	27.94	557.3641	−8.8	C_30_H_54_O_9_	359.1872	Rha-C_12:1_-C_12_	5(-2H)	5	
**12**	29.52	583.3678	−28.8	C_32_H_56_0_9_	387.2296	Rha-C_14:1_-C_12:1_	7(-2H)	5(-2H)	
**13**	29.84	559.3688	−28.2	C_30_H_56_O_9_	389.2431	Rha-C_14_-C_10_	7	3	
**14**	31.46	585.3919	−14.3	C_32_H_58_0_9_	415.2556	Rha-C_16:1_-C_10_	9(-2H)	3	
**15**	32.20	585.3919	−14.3	C_32_H_58_0_9_	387.2296	Rha-C_14:1_-C_12_	7(-2H)	5	
						Rha-C_12_-C_14:1_	5	7(-2H)	
**16**	35.85	587.4017	−24.2	C_32_H_60_0_9_	417.2728	Rha-C_16_-C_10_	9	3	

^a^ Rha denotes the a-L-rhamnopyranosyl moiety, the designation C_x_ means a fatty acid chain with chain length of X, C_x:1_ means a fatty acid chain with chain length of X and with one unsaturated bond (–2H).

**Table 2:** Relative abundance of the different rhamnolipid congeners in mixture detected in each growth condition.

	RL Relative Abundance (%)															
Carbon Source	1	2	3 *	4	5	6	7	8 *	9	10	11	12 *	13	14 *	15 *	16
**Monosaccharides**																
**Glucose**	10.4		1.6	15.1		23.4	25.1	3.4	8.5	1.4	2.8	0.2	6.2	0.6	0.2	1.1
**Mannose**	27.9	0.1	0.4	5.9		29.3	25.1	2.4	5.1	0.6	0.8	0.1	1.5	0.5		0.3
**Rhamnose**	13.2	0.6	0.2	6.1		42.9	32.4	1.2	2.4	0.6	0.4					
**Polysaccharides**																
**Starch**	29.6			9.2		36.4	19.8	0.6	2.6	0.2	0.3	0.9	0.4			
**Xylan**	18.9			9.7		50.1	18.8	0.9	1.6							
**Fatty acids and derivatives**																
**Glycerol**	5.2	0.2	5.3	9.5	0.5	19.1	14.0	8.0	12.5	1.9	5.0	0.3	11.8	4.7	1.0	1.0
**UCO**	5.9		3.2	13.4		17.4	17.0	10.7	5.4	2.6	6.4	1.3	12.2	3.3	1.0	0.2
**Petroleum derivatives**																
**Benzene**	24.1	0.3	0.2	6.4		37.3	25.5	2.0	2.0	0.3	0.6	1.3				
**Diesel**	22.7	0.6	2.2	10.8	0.5	30.4	22.8	1.9	2.7	0.8	1.1	0.3	2.7	0.5		
**PAHs**																
**Anthracene**	4.6			9.0	1.2	28.4	50.2	1.0	2.1	0.3	0.6		2.4	0.2		
**Phenanthrene**						100.0										
**Pyrene**																
**Miscellaneous**																
**TYP CE**	8.5	0.1	0.5	11.1	0.8	21.7	24.1	4.0	7.4	4.9	4.0	0.3	9.4	1.7	0.5	1.0
**TYP 80%**	10.4	2.5	2.1	12.5	1.3	16.9	22.2	6.2	7.8	2.0	4.2	0.1	10.7	0.9	0.1	0.1
**TYP 100%**				6.1	2.6	16.9	21.9	7.9	8.5	10.4	6.1	1.4	11.2	4.4	0.6	2.0
**Control**																

* New RLs found in this study.

**Table 3:** Antimicrobial activity of M15 MeOH fractions reported as minimum inhibitory concentration (MIC) value.

Minimum Inhibitory Concentration (µg/mL)							
Strains	Fractions			Strains	Fractions		
Gram-Positive	60%	80%	100%	Gram-Negative	60%	80%	100%
*B. cereus*	-	6.25	3.13	*S. maltophilia 700475*	-	12.5	25.0
*L. monocytogenes*	-	25.0	12.5	*S. maltophilia 13637*	-	12.5	12.5
*S. aureus 29213*	-	12.5	25.0	*S. maltophilia 13636*	-	12.5	25.0
*S. aureus 23235*	-	12.5	12.5	*A. baumannii*	-	-	-
*S. aureus 6538P*	-	25.0	25.0	*B. metallica*	-	-	-
*S. epidermidis*	-	50.0	100	*E. coli*	-	-	-
*S. xylosus*	-	-	-	*K. pneumoniae*	-	-	-
				*P. aeruginosa*	-	-	-
				*S*. Enteritidis	-	-	-
				*S*. Typhimurium	-	-	-

**Table 4:** Antimicrobial activity of different growth conditions crude extracts reported as MIC values.

Minimum Inhibitory Concentration (µg/mL)							
Strains	Glucose	Mannose	Rhamnose	Glycerol	TYP	Xylan	Starch
**Gram-positive**							
*B. cereus*	6.25	100	-	3.13	7.81	-	-
*L. monocytogenes*	25.0	-	-	3.13	62.5	-	-
*S. aureus 6538P*	37.5	100	-	6.25	98.3	-	-
*S. epidermidis*	-	-	-	3.13	62.5	-	-
**Gram-negative**							
*S. maltophilia 13637*	50.0	-	-	3.13	62.5	-	-

## Supplementary Materials

The following are available online at https://www.mdpi.com/1660-3397/18/5/269/s1, Figure S1: HRESI-MS spectrum of compound 1, Figure S2: HRESI-MS spectrum of compound 2, Figure S3: HRESI-MS spectrum of compound 3, Figure S4: HRESI-MS spectrum of compound 4, Figure S5: HRESI-MS spectrum of compound 5, Figure S6: HRESI-MS spectrum of compound 6, Figure S7: HRESI-MS spectrum of compound 7, Figure S8: HRESI-MS spectrum of compound 8, Figure S9: HRESI-MS spectrum of compound 9, Figure S10: HRESI-MS spectrum of compound 10, Figure S11: HRESI-MS spectrum of compound 11, Figure S12: HRESI-MS spectrum of compound 12, Figure S13: HRESI-MS spectrum of compound 13, Figure S14: HRESI-MS spectrum of compound 14, Figure S15: HRESI-MS spectrum of compound 15, Figure S16: HRESI-MS spectrum of compound 16, Carbon source influence on RLs production, Figure S17: Base peak chromatogram of anthracene extract, Figure S18: Base Peak chromatogram of benzene extract, Figure S19: Base peak chromatogram of control extract, Figure S20: Base Peak chromatogram of diesel extract, Figure S21: Base peak chromatogram of glucose extract, Figure S22: Base Peak chromatogram of glycerol extract, Figure S23: Base peak chromatogram of mannose extract, Figure S24: Base Peak chromatogram of used cooking oil extract, Figure S25: Base peak chromatogram of phenanthrene extract, Figure S26: Base Peak chromatogram of pyrene extract Figure S27: Base peak chromatogram of rhamnose extract, Figure S28: Base peak chromatogram of starch extract, Figure S29: Base peak chromatogram of xylan extract.
