# Supplementary material for: Characterization of a New Mixture of Mono-Rhamnolipids Produced by Pseudomonas gessardii Isolated from Edmonson Point (Antarctica)
Source: Mar Drugs. 2020 May 20;18(5):269. doi: 10.3390/md18050269 (PMC7281774; doi:10.3390/md18050269)

Characterization of a new mixture of mono-rhamnolipids produced by *Pseudomonas gessardii* isolated from Edmonson Point (Antarctica)

Carmine Buonocore [1], Pietro Tedesco [2], Fortunato Palma Esposito [3], Giovanni Andrea Vitale [1], Rosa Giugliano[4], Maria Chiara Monti [5], Maria Valeria D'Auria [6,*], Donatella de Pascale [1,3,*].

[1] Institute of Biochemistry and Cell Biology, National Research Council, Naples, Italy, carmine.buonocore@ibbc.cnr.it; giovanniandrea.vitale@ibbc.cnr.it;

[2] TBI-INSA, 135 Avenue de Rangueil, Toulouse, France; tedesco@insa-toulouse.fr

[3] Department of Marine Biotechnology, Stazione Zoologica Anton Dohrn, Villa Comunale Naples, 80125, Italy; fpefortunato@gmail.com, donatella.depascale@szn.it.

[4] Department of Experimental Medicine, University of Campania "Luigi Vanvitelli", Naples, Italy; rosa.giugliano@unicampania.it;

[5] Department of Pharmacy, University of Salerno, Salerno, Italy; mcmonti@unisa.it;

[6] Department of Pharmacy, University of Naples "Federico II", Naples, Italy; madauria@unina.it;

* Correspondence:

Dr. Donatella de Pascale, Department of Marine Biotechnology, Stazione Zoologica Anton Dohrn, Villa Comunale Naples, 80125, Naples, Italy. Tel.: +39 081 5833319 donatella.depascale@szn.ir

Prof. Maria Valeria D'Auria, Department of Pharmacy, University of Naples "Federico II", Naples, Italy; madauria@unina.it

Contents S2

Spectral data of rhamonolipids congeners
Figure S1. HRESI-MS spectrum of compound 1 S3
Figure S2. HRESI-MS spectrum of compound 2 S3
Figure S3. HRESI-MS spectrum of compound 3 S3
Figure S4. HRESI-MS spectrum of compound 4 S3
Figure S5. HRESI-MS spectrum of compound 5 S3
Figure S6. HRESI-MS spectrum of compound 6 S4
Figure S7. HRESI-MS spectrum of compound 7 S4
Figure S8. HRESI-MS spectrum of compound 8 S4
Figure S9. HRESI-MS spectrum of compound 9 S4
Figure S10. HRESI-MS spectrum of compound 10 S4
Figure S11. HRESI-MS spectrum of compound 11 S5
Figure S12. HRESI-MS spectrum of compound 12 S5
Figure S13. HRESI-MS spectrum of compound 13 S5
Figure S14. HRESI-MS spectrum of compound 14 S5
Figure S15. HRESI-MS spectrum of compound 15 S5
Figure S16. HRESI-MS spectrum of compound 16 S6
Carbon source influence on RLs production S6
Figure S17. Base Peak chromatogram of anthracene extract S6
Figure S18. Base Peak chromatogram of benzene extract S6
Figure S19. Base Peak chromatogram of control extract S7
Figure S20. Base Peak chromatogram of diesel extract S7
Figure S21. Base Peak chromatogram of glucose extract S7
Figure S22. Base Peak chromatogram of glycerol extract S7
Figure S23. Base Peak chromatogram of mannose extract S8
Figure S24. Base Peak chromatogram of S.E. oil extract S8
Figure S25. Base Peak chromatogram of phenanthrene extract S8
Figure S26. Base Peak chromatogram of pyrene extract S8
Figure S27. Base Peak chromatogram of rhamnose extract S9
Figure S28. Base Peak chromatogram of starch extract S9
Figure S29. Base Peak chromatogram of xylan extract S9

Spectral data of rhamonolipids congeners

Figure S1. HRESI-MS spectrum of compound 1

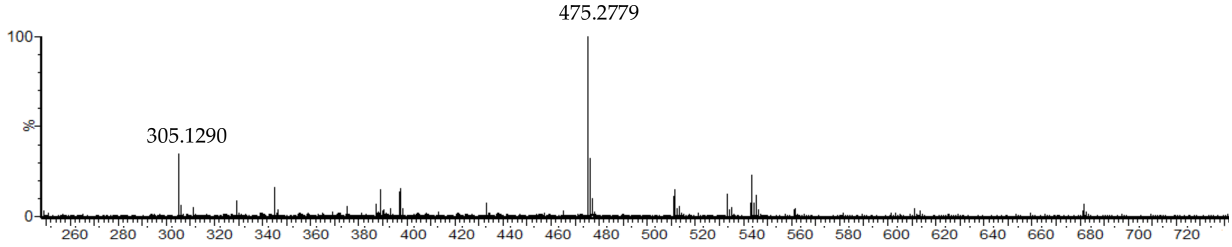

Figure S2. HRESI-MS spectrum of compound 2

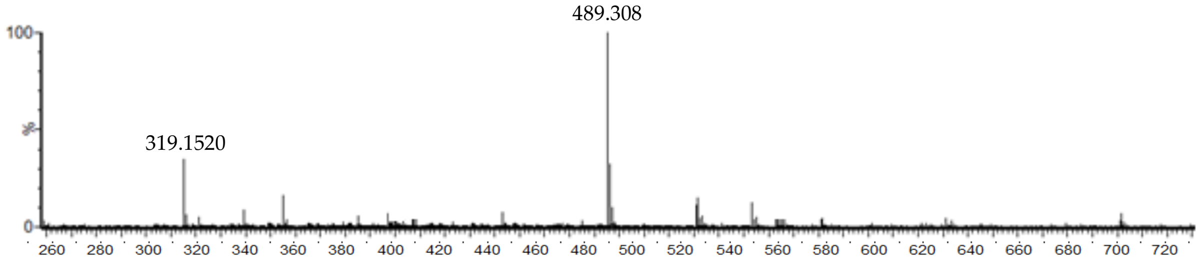

Figure S3. HRESI-MS spectrum of compound 3

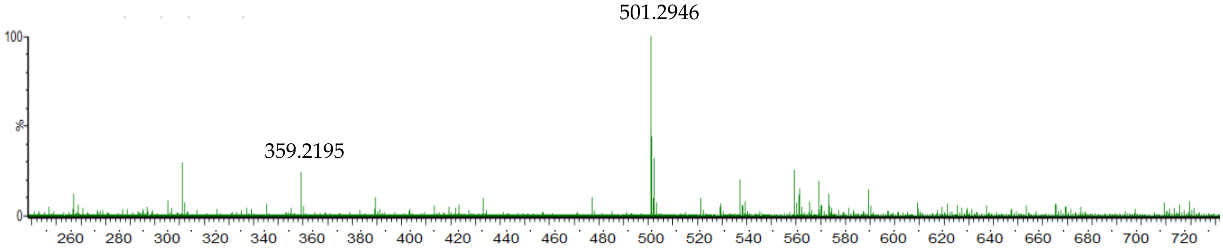

Figure S4. HRESI-MS spectrum of compound 4

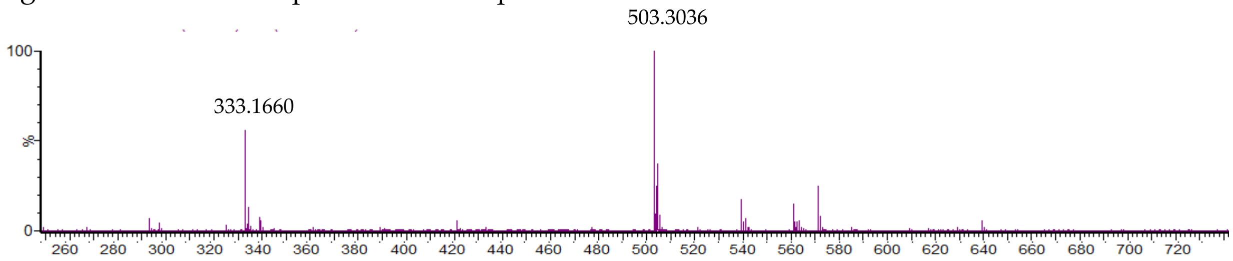

Figure S5. HRESI-MS spectrum of compound 5

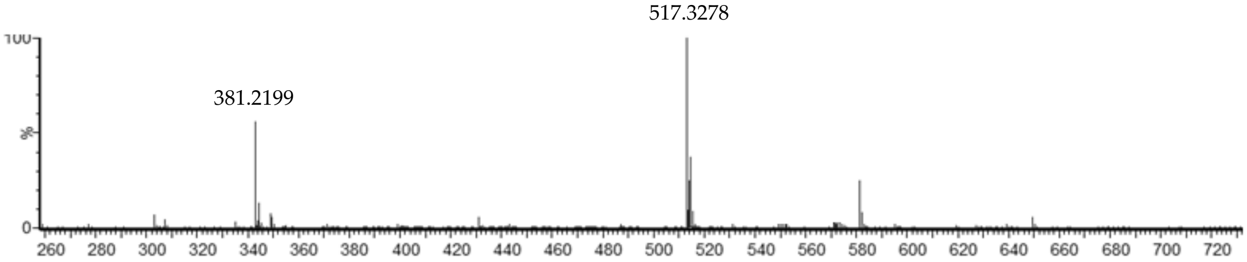

Figure S6. HRESI-MS spectrum of compound 6

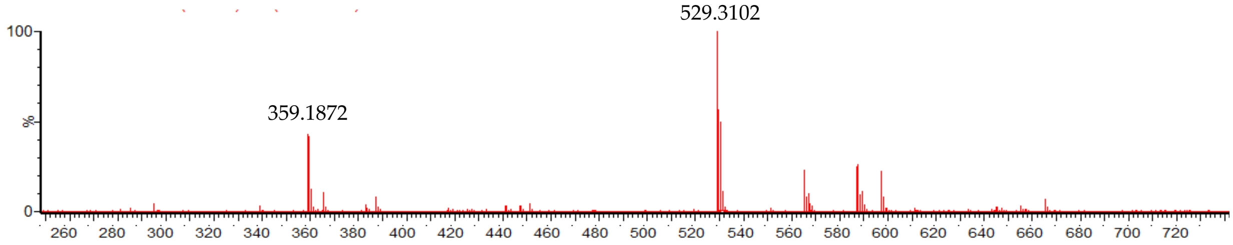

Figure S7. HRESI-MS spectrum of compound 7

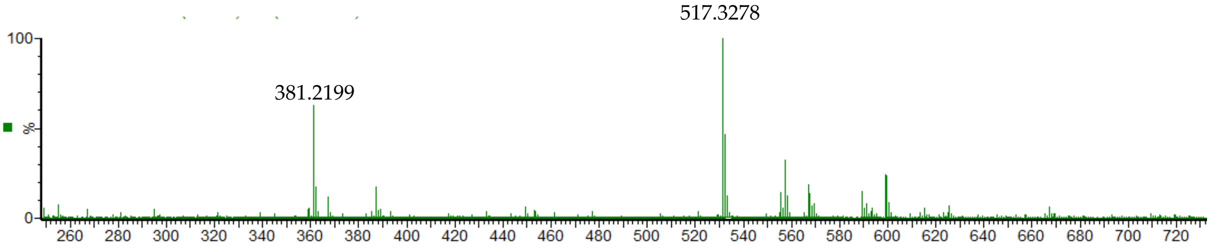

Figure S8. HRESI-MS spectrum of compound 8

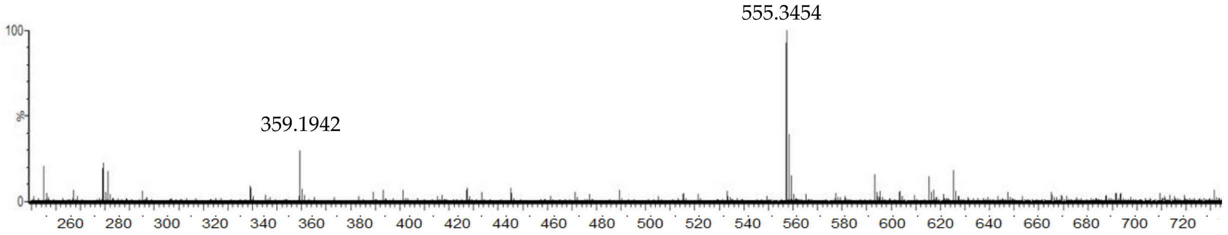

Figure S9. HRESI-MS spectrum of compound 9

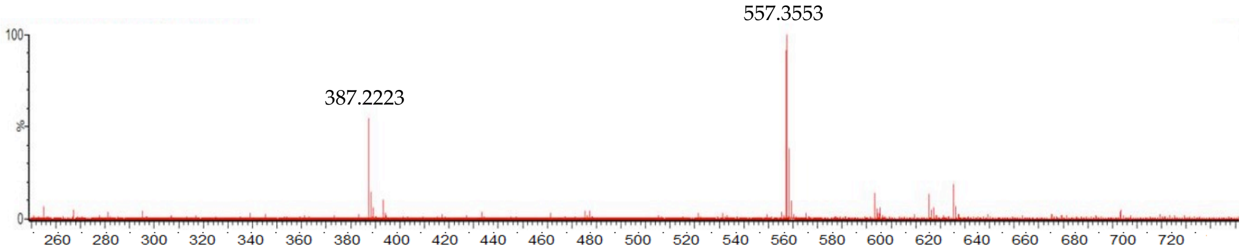

Figure S10. HRESI-MS spectrum of compound 10

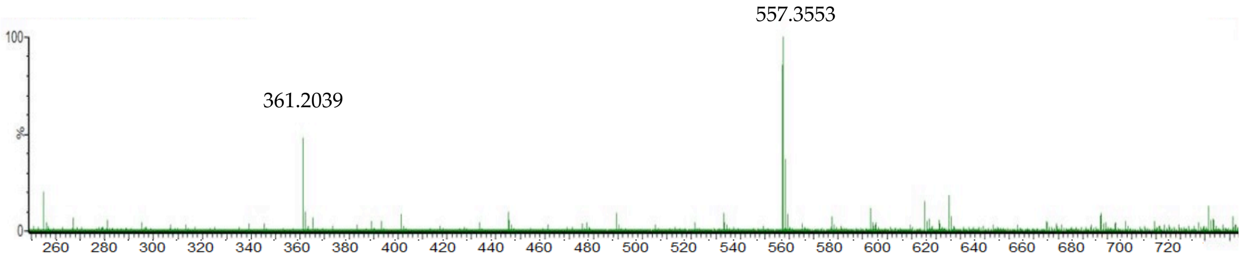

Figure S11. HRESI-MS spectrum of compound 11

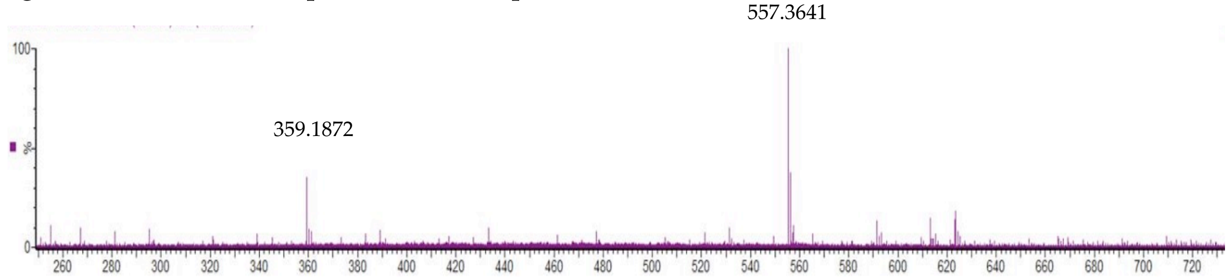

Figure S12. HRESI-MS spectrum of compound 12

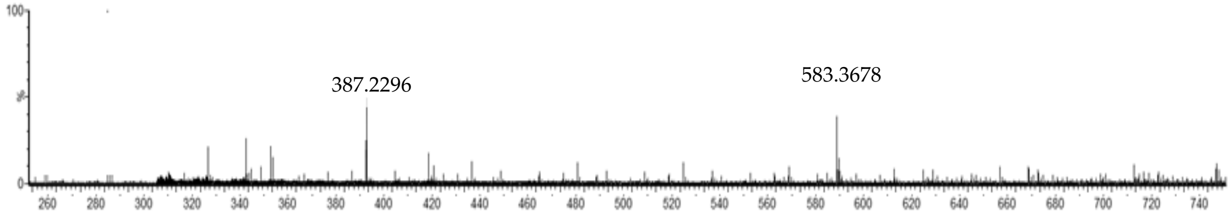

Figure S13. HRESI-MS spectrum of compound 13

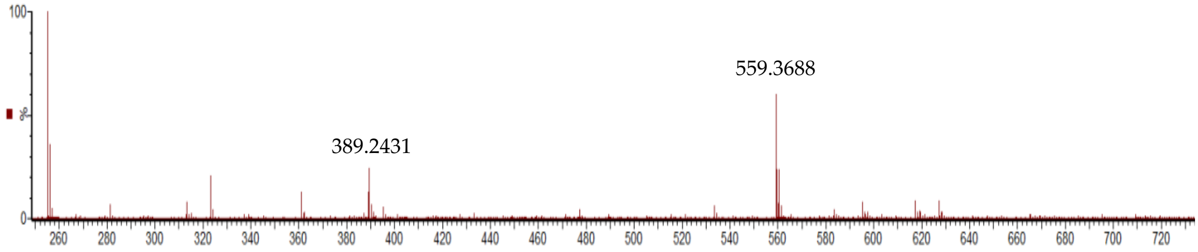

Figure S14. HRESI-MS spectrum of compound 14

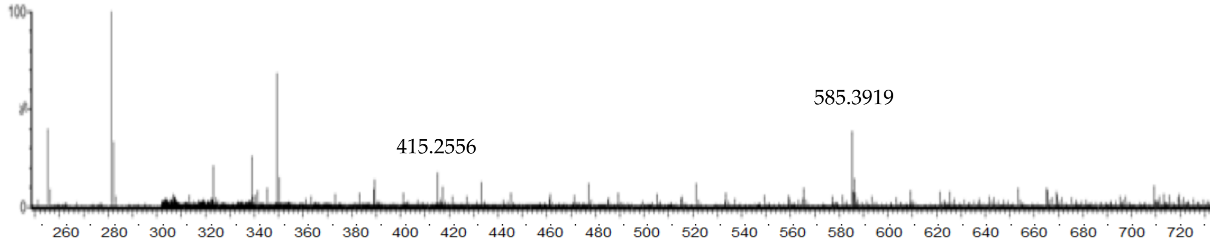

Figure S15. HRESI-MS spectrum of compound 15

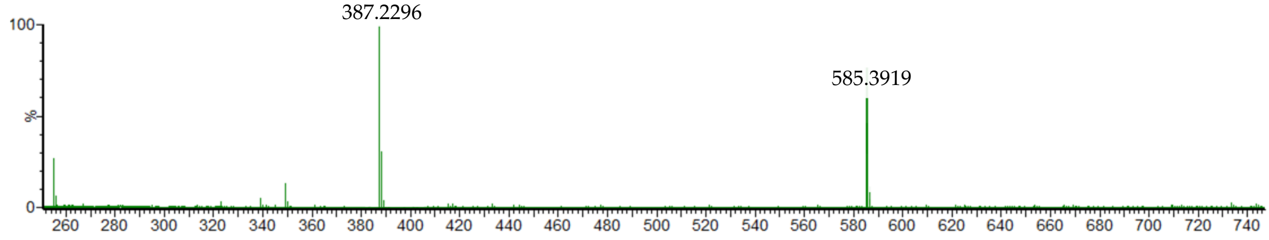

Figure S16. HRESI-MS spectrum of compound 16

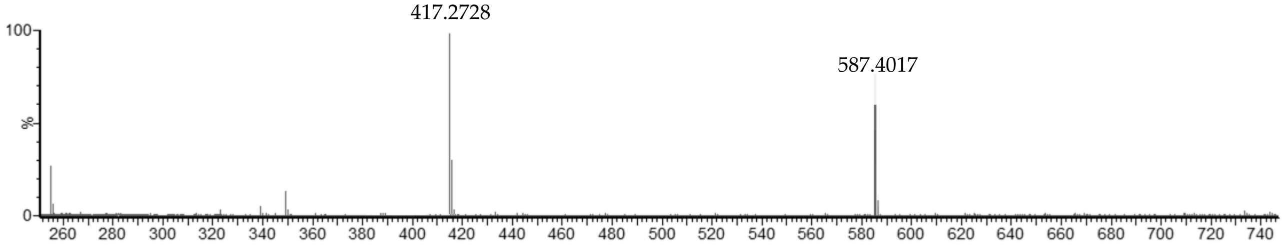

Carbon source influence on RLs production

Figure S17. Base Peak chromatogram of anthracene extract

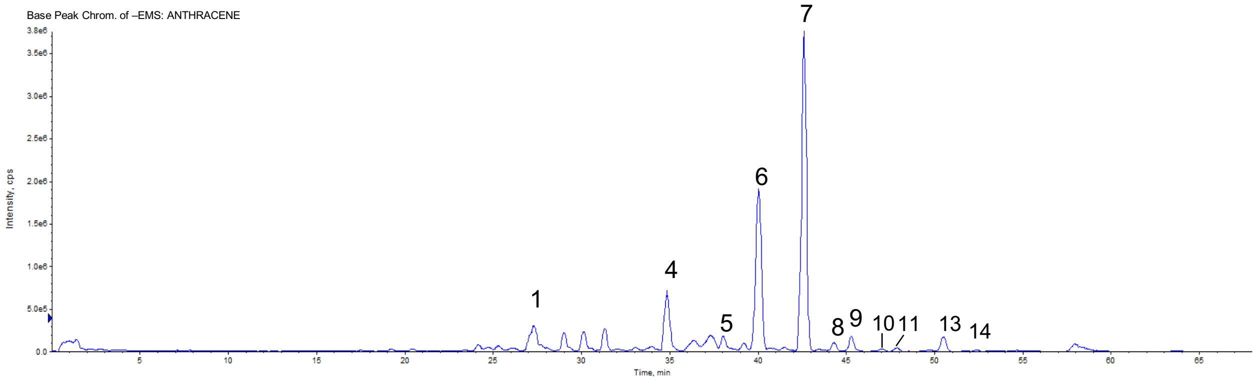

Figure S18. Base Peak chromatogram of benzene

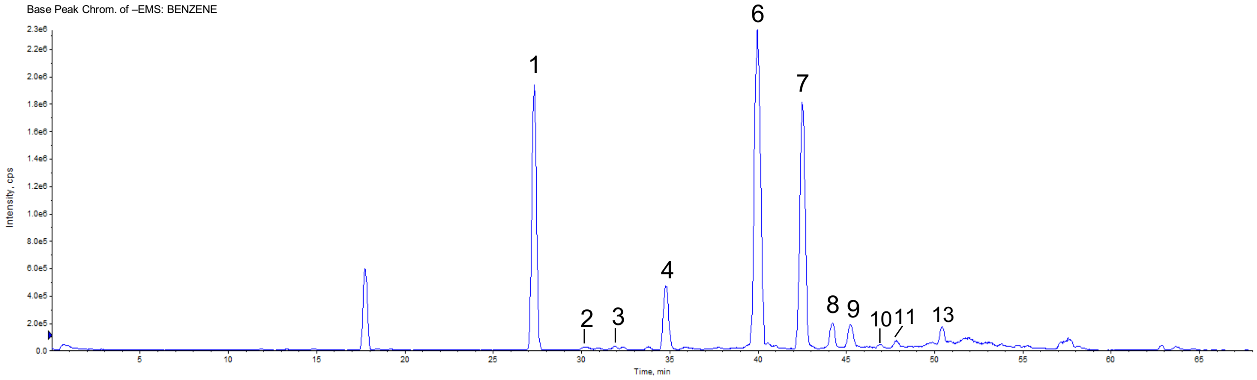

Figure S19. Base Peak chromatogram of control extract

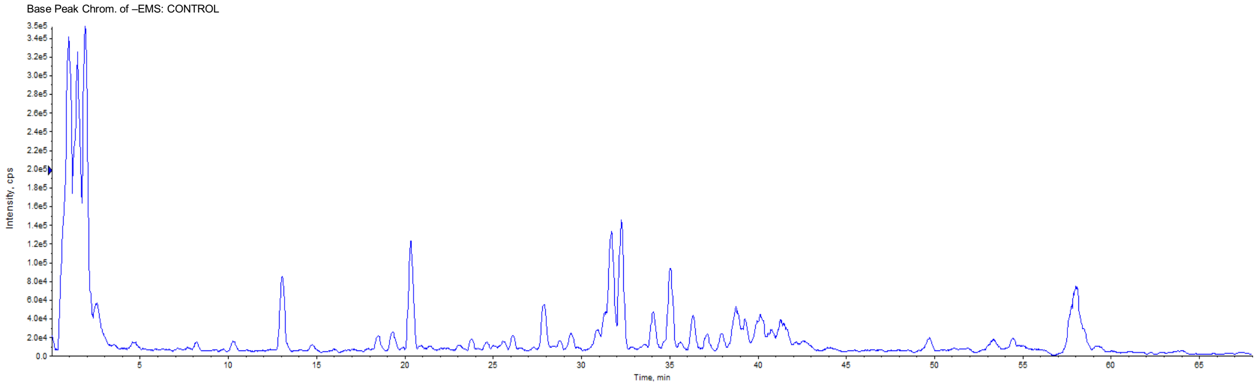

Figure S20. Base Peak chromatogram of diesel extract

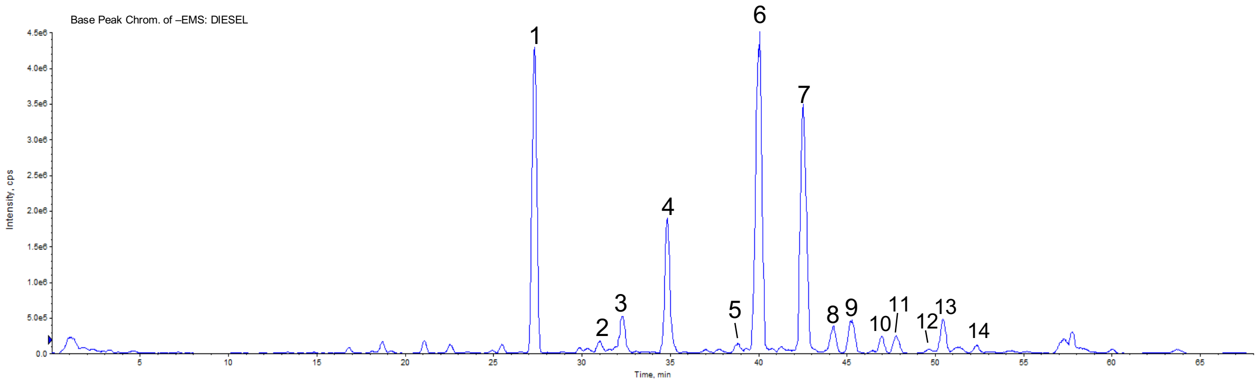

Figure S21. Base Peak chromatogram of glucose extract

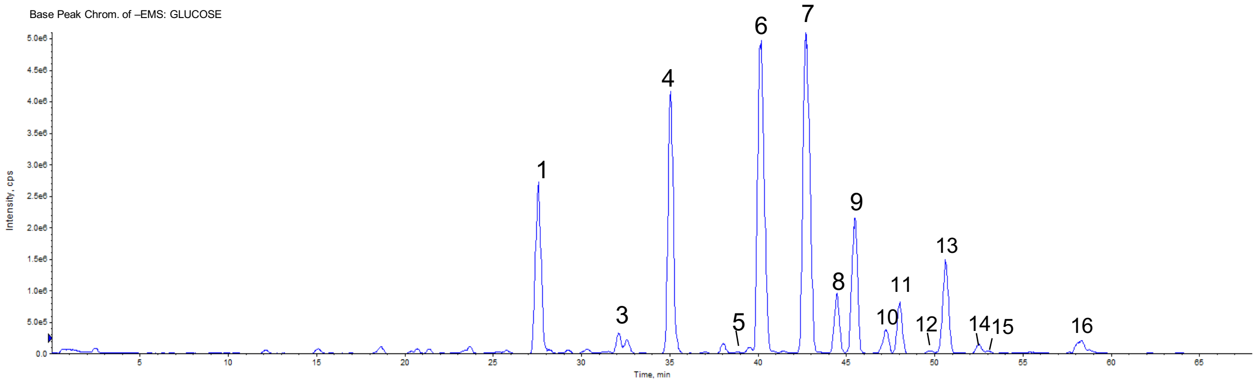

Figure S22. Base Peak chromatogram of glycerol extract

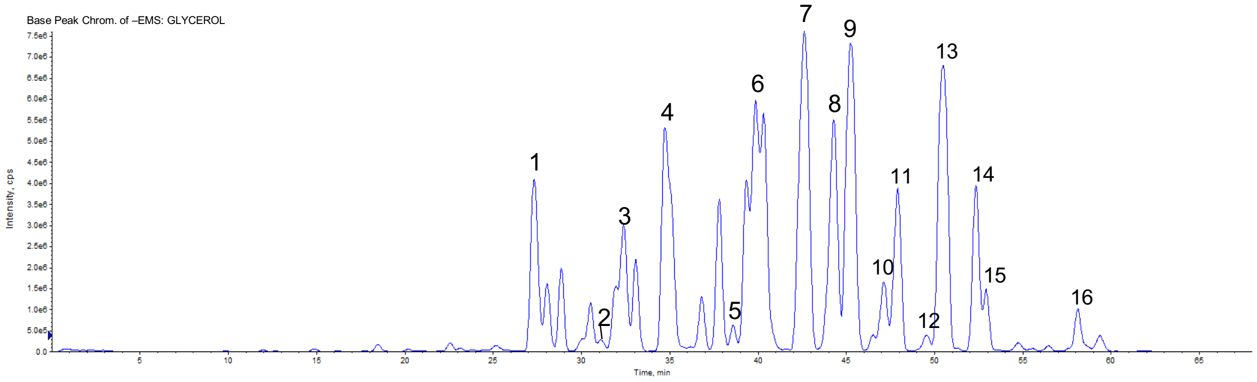

Figure S23. Base Peak chromatogram of mannose extract

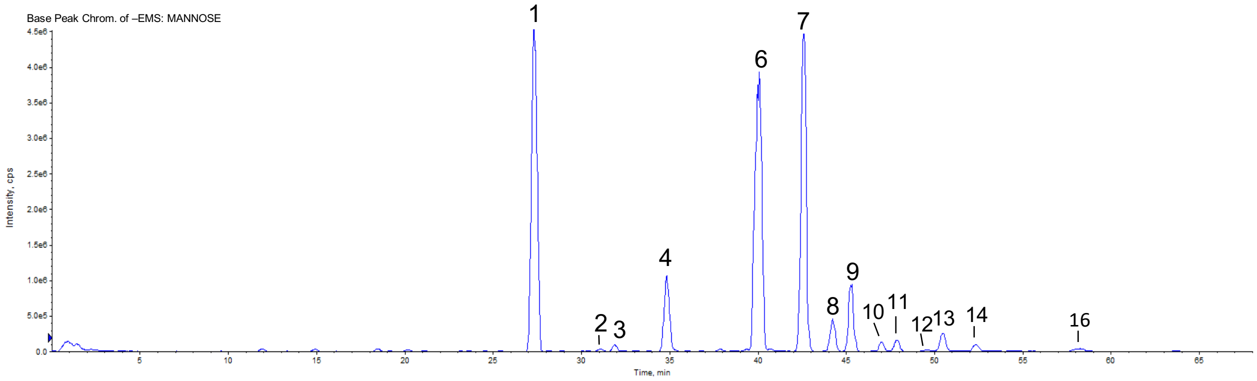

Figure S25. Base Peak chromatogram of used cooking oil extract

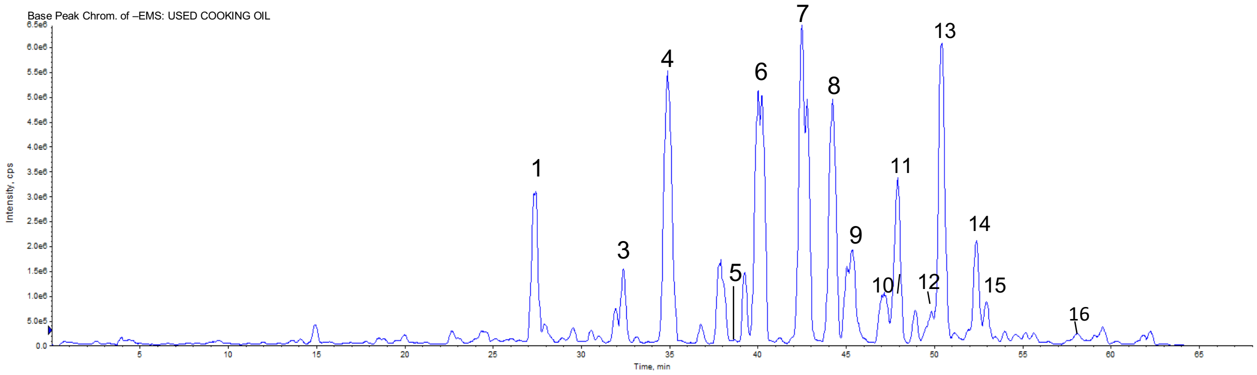

Figure S25. Base Peak chromatogram of phenanthrene extract

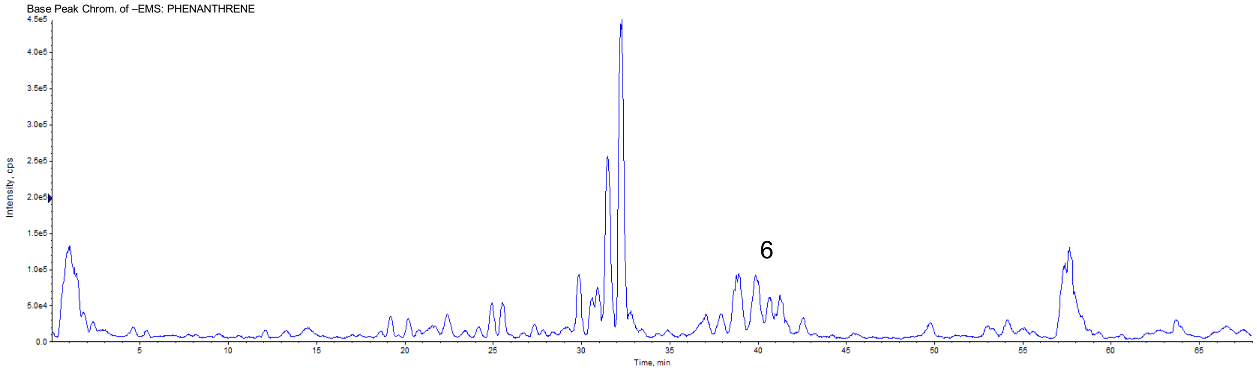

Figure S26. Base Peak chromatogram of pyrene extract

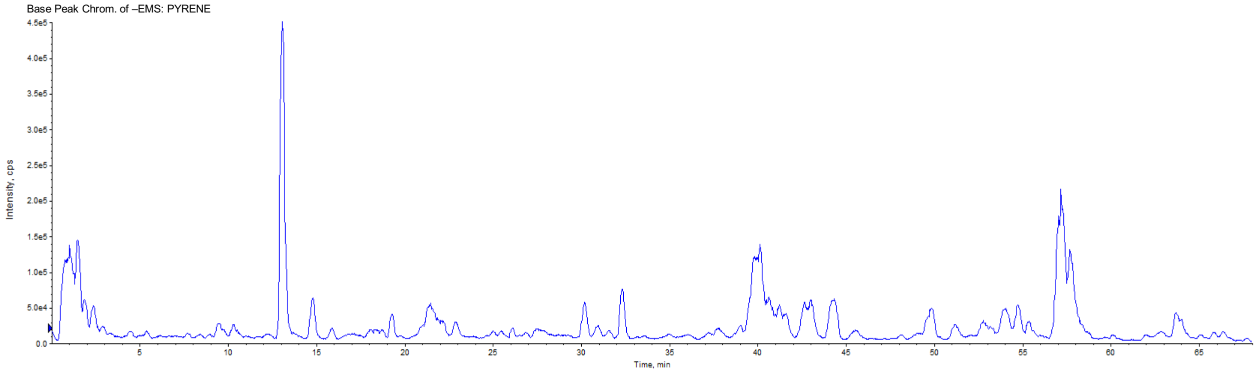

Figure S27. Base Peak chromatogram of rhamnose extract

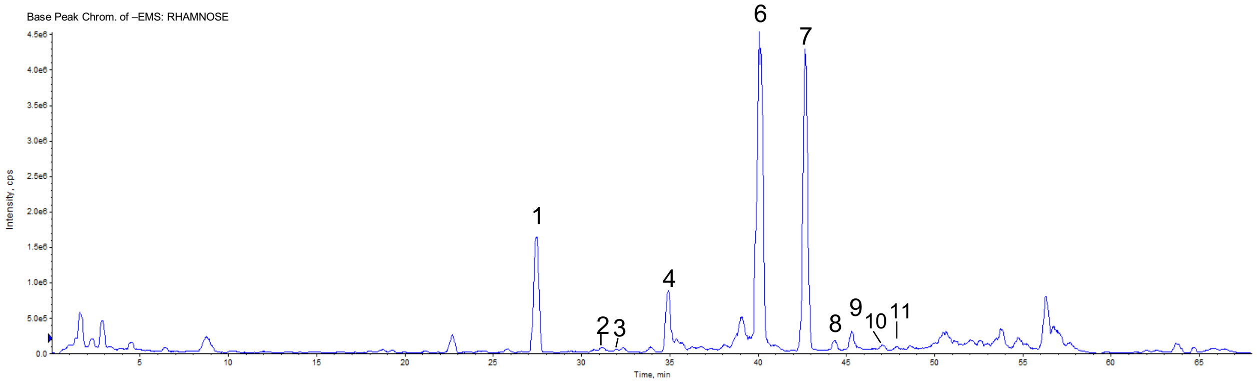

Figure S28. Base Peak chromatogram of starch extract

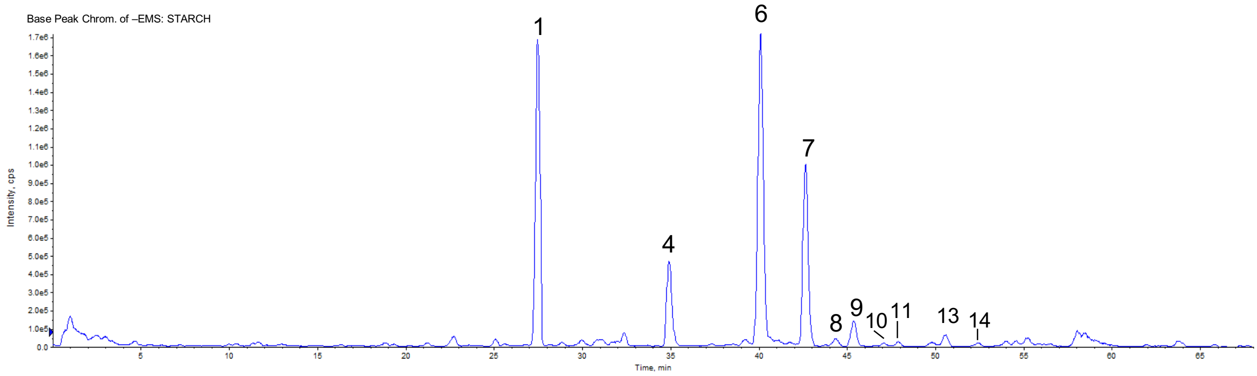

Figure S29. Base Peak chromatogram of xylan extract

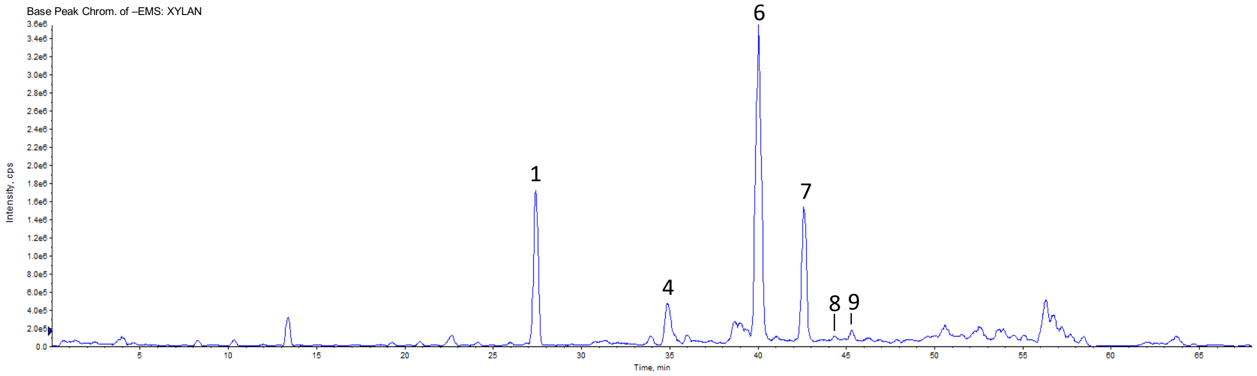

Supplement: Supplementary file 1 [file marinedrugs-18-00269-s001.pdf]
